# Supplementary material for: Social support and risk perception of influenza among Chengdu residents: A cross-sectional study during post-pandemic recovery
Source: PLoS One. 2025 Aug 29;20(8):e0331052. doi: 10.1371/journal.pone.0331052 (PMC12396661; doi:10.1371/journal.pone.0331052)
Supplement: Attachment — Submitted filename: PONE_Feng.docx [file pone.0331052.s001.docx]

**Manuscript title:** Social Support and Risk Perception of Influenza Among Chengdu Residents: A Cross-Sectional Study During Post-Pandemic Recovery

**Comments to the Editor:**

Dear Editor,

Thank you for giving me the opportunity to review this manuscript.

The manuscript entitled above describes a cross-sectional study to investigate the interplay between social support and influenza risk perception post COVID among residents in Chengdu, China. There are some points that need to be addressed and revised by the authors first before finally accepting the manuscript for publication.

Best Regards,

**General Comments:**

Overall, the authors need to expand on their methodology and how the scores were calculated. The study has multiple design flaws, see specific comments below.

**Specific Comments:**

Methods:

1. The authors mention using convenient sampling but fail to report where the sample was drawn from.
2. Who is the population with access to the questionnaire star platform?
3. Does including non-adults (age criteria 12-70) affect / result in increase variability in the risk perception scale results?
4. Was the risk perception scale validated for children and adults?

Results:

1. Table 1: 65.7% were < 25, 28% are 25-40, and 6.5 % > 40; do the author thinks the skewed results favoring your participants could have skewed the results. Since study was planned for age 12-70.
2. I am not sure how the risk score was calculated (the percentage).
3. Same comment for the social support score.

Limitation:

1. Authors mention over representation of healthcare professionals but it seems to me like we are mainly comparing healthcare professionals to students (two major groups) which could be misleading are the two groups have major differences in age, education, and life experiences.
2. All other groups lumped in others are a small percentage, raising concerns about the results.

Minor comments:

1. Further details about the validation of the risk perception scale need to be added in addition to the reference.
