# Supplementary material for: Social support and risk perception of influenza among Chengdu residents: A cross-sectional study during post-pandemic recovery
Source: PLoS One. 2025 Aug 29;20(8):e0331052. doi: 10.1371/journal.pone.0331052 (PMC12396661; doi:10.1371/journal.pone.0331052)
Supplement: Attachment — Submitted filename: Response to Reviewers.doc [file pone.0331052.s002.doc]

**Response to Reviewers**

Dear Dr. Xing-Xiong An and Reviewers,

Thank you for your thoughtful feedback on our manuscript titled "Social Support and Risk Perception of Influenza Among Chengdu Residents: A Cross-Sectional Study During Post-Pandemic Recovery." We appreciate the reviewers’ constructive comments and have carefully addressed each concern. Below is a point-by-point response to the reviewers’ suggestions. Revisions in the manuscript are highlighted in the tracked-changes version.

**Journal Requirements:**

**1. When submitting your revision, we need you to address these additional requirements.Please ensure that your manuscript meets PLOS ONE's style requirements, including those for file naming.**

Thank you for your feedback. We have revised our manuscript in accordance with PLOS ONE's style requirements. This includes adjustments to the title format, font sizes, and reference list, as well as ensuring proper file naming conventions. We have made comprehensive changes throughout the document to ensure full compliance with the journal’s guidelines.

Thank you for your attention to this matter.

**2.We note that you have indicated that there are restrictions to data sharing for this study. For studies involving human research participant data or other sensitive data, we encourage authors to share de-identified or anonymized data. However, when data cannot be publicly shared for ethical reasons, we allow authors to make their data sets available upon request.**

Thank you for your guidance regarding data sharing. We understand the importance of making our data accessible while ensuring the privacy and confidentiality of our participants. For our study, We have deposited the de-identified dataset for our study in Zenodo, a free and widely recognized multidisciplinary repository (Lines 252-254).

Repository: Zenodo

DOI: [[10.5281/zenodo.15389586](https://doi.org/10.5281/zenodo.15389586)]

Link: https://zenodo.org/records/15389587

This repository ensures the data is accessible and preserved in accordance with best practices.

**Reviewer comments:**

**Reviewer #1**

**Major Issues:**

**1. Sampling Problems:**

Thank you for highlighting this critical limitation. We have revised the Limitations section to explicitly address the demographic skew in our sample (young age, female predominance, and healthcare worker overrepresentation) (Lines 209-217). We elaborated on how these factors might bias risk perception outcomes and reduce the generalizability of results. Specifically, we acknowledged that healthcare workers’ desensitization, coupled with age- and gender-specific behavioral patterns, could limit the applicability of findings to the general public. We also emphasized the need for future studies to adopt more representative sampling frameworks. These revisions strengthen the transparency of methodological constraints and align with best practices in reporting epidemiological studies.

**2. Risk Perception Tool Not Fully Explained:**

We sincerely thank the reviewers for their constructive feedback. In the article (Lines 121-127), we explain in detail the development and validation process of the Risk Perception Scale (RPS) : including scale development, validation process (content validity, structural validity, reliability) and the alignment with standards. Thank you for emphasizing this oversight. We believe that the increased clarity has enhanced the rigor of our research methods.

**3. Weak Correlation :**

We are grateful for the profound insights of the reviewers. As suggested, we have revised the discussion section to clearly address the actual meaning of weak correlation and the nuances of the context. The main new additions include:

3.1 Clarification of effect size: We emphasize that although the negative correlation between social support and risk perception is statistically significant (p < 0.01), the smaller effect size (r = -0.125) indicates that the real-world impact is limited (Lines 188-195).

3.2 Contextual explanation: We introduced cultural factors (such as the self-reliant norms of society) and structural differences in support types (subjective and objective support) as reasonable explanations for the weak association. These supplementary contents refer to the recent literature on the influence of culture on healthy behaviors (Lines 188-195)..

3.3 A more detailed table: We have presented in detail the multi-dimensional results of the correlation analysis between public social support and risk perception (Table 5).

These revisions appear in the updated manuscript. We believe this strengthens the interpretation of the results and is consistent with the valuable feedback from the reviewers.

**4. Missing Theory:**

Thank you very much for your insightful comments on our manuscript. We have carefully considered your suggestions and have revised our manuscript accordingly.

We have incorporated the Health Belief Model (HBM) as the theoretical framework to explain the relationship between social support and risk perception in our study. The HBM posits that health behavior decisions are driven by four dimensions of cognition: threat appraisal (perceived severity and susceptibility), behavioral appraisal (benefits and barriers), and external regulatory factors (cues to action and self-efficacy). This model provides a comprehensive framework to understand how social support can influence risk perception and subsequent health behaviors. In our revised manuscript, we have explicitly discussed how social support can influence risk perception through multiple pathways as suggested by the HBM. Specifically, we have highlighted the following points:

4.1 Informational Support as Cues to Action: Informational support from authoritative sources can serve as cues to action, directly triggering preventive behaviors. In our study, we found that objective support (e.g., access to healthcare resources) was positively correlated with risk susceptibility perception, suggesting that individuals with higher objective support were more aware of their vulnerability to influenza (Lines 57-66).

4.2 Instrumental and Emotional Support: Instrumental support (e.g., practical assistance) and emotional support (e.g., psychological comfort) can reduce perceived barriers and enhance self-efficacy, indirectly promoting health behaviors. Our results showed that subjective support (e.g., familial care) was higher than objective support, indicating that emotional and social connections play a significant role in shaping individuals' risk perception (Lines 64-66).

4.3 Overall Social Support and Risk Perception: We found a weak inverse correlation between total social support and risk perception, suggesting that higher levels of social support may reduce the perceived threat of influenza. This aligns with the HBM's framework, where external cues and support systems can mitigate perceived barriers and enhance preventive behaviors.

By integrating the HBM into our study, we have provided a clearer explanation of the mechanisms through which social support influences risk perception. We believe that this theoretical grounding enhances the robustness and interpretability of our findings.We have revised the relevant sections of our manuscript to reflect these changes and have included detailed explanations and references to the HBM throughout the text (Lines 196-201).

Thank you again for your valuable feedback. We look forward to your positive response.

**5. Too Focused on p-values::**

Thank you for your valuable feedback on emphasizing effect sizes and clinical significance. We have revised the manuscript as follows:

5.1 Effect Size Reporting in Results: Table 5 now explicitly labels Spearman’ s r values and includes a footnote defining effect size magnitudes (small, medium, large) per Cohen’ s criteria. This clarifies the practical relevance of the correlations.

5.2 Clinical vs. Statistical Significance in Discussion: A new paragraph (Lines 193-201) discusses the small effect sizes observed, distinguishing statistical significance from clinical relevance. This aligns with your suggestion and strengthens the interpretation of findings.

These revisions enhance transparency and provide a balanced interpretation of both statistical and practical significance. We appreciate your guidance in improving the manuscript’ s rigor.

**6. Minor Issues:**

**6.1 English and Grammar:**

Thank you for your feedback. We have thoroughly revised the manuscript with the assistance of a professional English editor to enhance clarity, correct grammatical errors, and ensure consistent terminology. All sections now adhere to academic writing standards.

**6.2 Missing or Unclear Tables:**

We apologize for the formatting inconsistencies. All tables have been reformatted for clarity, with proper alignment, headers, and annotations. Tables are now fully integrated into the text and comply with journal guidelines.

**3. Ethics Approval Numbers:**

Thank you for noting this oversight. The ethics approval number has been unified to "WCSU-2022-787" across all sections, ensuring consistency (Lines 91-92).

**4. Duplicate References:**

We appreciate your careful review. Duplicate references have been removed, and the list is now fully sequential and consistent with in-text citations.

**Reviewer #2**

**Major Issues:**

**1. Clarify Scale Interpretation:**

Thank you for your feedback on the need for a clearer explanation of the scale. We have made the following changes to the original manuscript:

Explanation of newly added scale scoring: Detailed scale information has been added to the introductory sections of SSRS and RPS, including scoring methods, scoring thresholds, classification criteria, etc., as well as cut-off points for specific dimensions. To ensure that readers can understand the scores reported in the results, these changes have increased the transparency of the manuscript and allowed for a more detailed interpretation of the research findings. Thank you very much for your constructive suggestions (Lines 102-127).

**2. Sampling Limitation**

Thank you for highlighting this critical limitation. We have revised the Limitations section to explicitly address the demographic skew in our sample (young age, female predominance, and healthcare worker overrepresentation) (Lines 209-230). We elaborated on how these factors might bias risk perception outcomes and reduce the generalizability of results. Specifically, we acknowledged that healthcare workers’ desensitization, coupled with age- and gender-specific behavioral patterns, could limit the applicability of findings to the general public. We also emphasized the need for future studies to adopt more representative sampling frameworks. These revisions strengthen the transparency of methodological constraints and align with best practices in reporting epidemiological studies.

**3. Discussion Depth**

Thank the reviewers for their profound insights. As suggested, we have delved deeply into the negative correlation between social support and risk perception, as well as the role of cultural self-reliance therein, to clearly explain the connection between the two. The newly added main contents include (Lines 188-201):

3.1 Clarification of effect size: We emphasize that although the negative correlation between social support and risk perception is statistically significant (p < 0.01), the smaller effect size (r = -0.125) indicates that the real-world impact is limited.

3.2 Contextual explanation: We introduced cultural factors (such as the self-reliant norms of society) and structural differences in support types (subjective and objective support) as reasonable explanations for the weak association. These supplementary contents refer to the recent literature on the influence of culture on healthy behaviors.

**4. Figures/Tables**

Thank you for your valuable feedback and suggestion regarding the addition of a visual summary of the correlation analysis in our manuscript. We have taken your advice into careful consideration and have incorporated a heatmap to visually represent the correlation analysis between social support and risk perception (Fig 1).

We have generated a heatmap using the data from our correlation analysis, which provides a clear and concise visual representation of the relationships between different dimensions of social support and risk perception. The heatmap highlights the significant correlations with appropriate annotations and color gradients, making it easier for readers to quickly grasp the key findings of our analysis.The heatmap has been included in the revised version of our manuscript as Figure 1. We believe that this visual summary complements the textual description of our results and enhances the overall presentation of our study.

We have also ensured that the heatmap adheres to the formatting and quality standards required by the journal, including the use of Arial font, appropriate font sizes, and high-resolution TIFF format for the image.

We hope that this addition will provide a more comprehensive understanding of our findings and improve the readability of our manuscript. Please let us know if there are any further suggestions or changes that you would like us to make.

**5. English Editing**

Thank you for your feedback. We have thoroughly revised the manuscript with the assistance of a professional English editor to enhance clarity, correct grammatical errors, and ensure consistent terminology. All sections now adhere to academic writing standards.

**Reviewer #3**

Thank you for your detailed and constructive feedback on our manuscript. We have carefully reviewed your comments and have made the necessary revisions to address each point. Below is our response to the specific issues you raised:

**1. Methods:
1.1 The authors mention using convenient sampling but fail to report where the sample was drawn from.**

We have clarified the sampling locations in the revised manuscript. The sample was drawn from seven highly populated urban districts in Chengdu, including Jinjiang District and Wuhou District. These districts were chosen to capture a diverse range of social groups. We have added this information to the "Study Design and Participants" section (Lines 83-88). **1.2 Who is the population with access to the questionnaire star platform?**

The population with access to the WJX platform includes residents of Chengdu aged 12-70 years who are literate in Mandarin and voluntarily participated in the study. We have specified this in the revised manuscript under the "Study Design and Participants" section (Lines 93-98). **1.3 Does including non-adults (age criteria 12-70) affect / result in increase variability in the risk perception scale results?**

1.3.1 The risk perception scale shows high structural validity and reliability in both the adolescent (12-18 years old) and adult (18-70 years old) groups (Lines 108-110). This point has been raised by us in the RPS introduction section. In order to quickly collect a large number of samples, the age range of the research subjects in this study was set as 12-70 years old. However, in the actual collected samples, there were only 8 non-adults (aged 12-17), accounting for 1%. The statistical results showed that there was no statistically significant difference in the median score of the risk perception scale between non-adults and adults (p>0.05). However, due to the small sample size of non-adults, it may lead to deviations in the research results. Therefore, we have added the discussion of this issue in the "Research Limitation" section (Lines 209-217).

1.3.2 We have conducted additional analyses to assess the impact of including non-adults (ages 12-17) on the variability of the risk perception scale results (Table 4). Our analysis shows that the inclusion of this age group did not significantly increase the variability of the results. The median risk perception scores for the different age groups were as follows: <25 years (37.00, IQR 33.00-41.00), 25-30 years (34.00, IQR 30.00-39.00), 30-35 years (35.00, IQR 29.50-40.00), 35-40 years (33.00, IQR 28.00-37.00), and ≥40 years (37.50, IQR 34.00-41.00). The Kruskal-Wallis test showed statistically significant differences among the age groups (p<0.001), but the effect sizes were small, indicating that the variability is within an acceptable range. We have included these details in the "Results" section and discussed the implications in the "Discussion" section (Lines 188-195).
**1.4 Was the risk perception scale validated for children and adults?**

The Risk Perception Scale (RPS) was developed based on a comprehensive literature review and theoretical framework to ensure cultural specificity and multidimensional risk structure. The scale was validated through a multi-stage process, including content validity assessment by 24 public health and psychology experts, exploratory analysis, and reliability testing. The validation process included individuals aged 12-70 years, ensuring that the scale is appropriate for both children and adults. We have detailed the validation process in the "Instruments" section and provided additional references to support the scale's validity across different age groups (Lines 111-127).

We have revised the relevant sections of our manuscript to reflect these changes and have included detailed explanations and references. Please find the revised manuscript attached for your review.

**2. Results:
2.1 Table 1: 65.7% were < 25, 28% are 25-40, and 6.5 % > 40; do the author thinks the skewed results favoring your participants could have skewed the results. Since study was planned for age 12-70.**

We acknowledge the demographic imbalance in our sample, with a higher proportion of younger participants. This skewed distribution was primarily due to the recruitment through academic and medical institutions in Wuhou District, which resulted in an overrepresentation of younger individuals. While this imbalance may influence the results to some extent, our analysis shows that the risk perception scores and social support scores still provide valuable insights across different age groups.

To address this concern, we have conducted additional subgroup analyses to compare the risk perception and social support scores across different age groups (Table 4). The results show that while younger adults (<25 years) and older adults (≥40 years) demonstrated higher risk perception, the overall trends and correlations remain consistent across the age spectrum. We have included these subgroup analyses in the revised manuscript and discussed their implications in the "Discussion" section.

Besides, we've taken the Limitations section and made it clearer about the differences in the people in our sample (like their age, the number of women, and how many healthcare workers there are) (Lines 209-230). We talked about how these things might make it hard to see the bigger picture and how the results might not be as useful as they could be. We totally get that age- and gender-specific behavioural patterns might mean that our findings can't be applied to everyone. We also emphasised how important it is for future studies to adopt more representative sampling frameworks. We've made some changes to make things clearer about the methodological constraints and we've followed the best ways of reporting epidemiological studies. **2.2 I am not sure how the risk score was calculated (the percentage).**

The risk perception score is calculated based on the Risk Perception Scale (RPS), which consists of 18 items and covers four dimensions: risk familiarity (6 items), risk controllability (4 items), risk severity (5 items), and risk susceptibility (3 items). Each item was scored using a 5-level Likert scale (1= strongly disagree to 5= strongly agree), with a total score ranging from 18 to 90 points. The score rate is calculated as the possible percentage of the actual score to the total score, that is, (actual score/highest score)×100%. For example, the score rate corresponding to a median score of 36 points is 40.00% (36/90×100%). We have added a detailed explanation of the scoring method in the "Instruments" section and provided an example calculation to clarify the process (Lines 111-127).
**2.3 Same comment for the social support score.**

The social support scores were calculated using the Social Support Rating Scale (SSRS), which includes 10 standardized items across three dimensions: objective support (2 items), subjective support (4 items), and support utilization (4 items). The scoring rules are as follows:

Items 1-3 and 6-10 use a 4-point rating scale (A-D options), with corresponding scores of 1-4, reflecting the support level from "none" to "full support."

Key items 4-5 use a source count method: "no sources" scores 0, and "the following sources" are scored based on the actual number of sources (each source scores 1). The total score ranges from 10 to 66. The score rate is calculated as the percentage of the total possible score, i.e., (actual score / maximum score) × 100%. For example, a median score of 37 corresponds to a score rate of 63.79% (37/58 × 100%).

We have added a detailed explanation of the scoring method for the SSRS in the "Instruments" section and provided an example calculation to clarify the process.

We have revised the relevant sections of our manuscript to reflect these changes and have included detailed explanations and examples to clarify the scoring methods. Please find the revised manuscript attached for your review (Lines 102-110).

**3. Limitation:
3.1 Authors mention over representation of healthcare professionals but it seems to me like we are mainly comparing healthcare professionals to students (two major groups) which could be misleading are the two groups have major differences in age, education, and life experiences.**

We agree that the over-representation of healthcare professionals and students in our sample could introduce biases and limit the generalizability of our findings. Healthcare professionals and students indeed differ significantly in terms of age, education, and life experiences, which may affect their risk perception and social support levels.

To address this concern, we have conducted additional analyses to compare the risk perception and social support scores between healthcare professionals and students separately (Table 4). The results show that while healthcare professionals exhibited lower risk perception than students, the differences were not solely due to age or education. We have included these subgroup analyses in the revised manuscript and discussed their implications in the "Discussion" section.

Additionally, we have emphasized in the "Limitations" section that the over-representation of these two groups may limit the generalizability of our findings to the broader population (Lines 209-217). We suggest that future studies should use more representative sampling methods to include a wider range of occupations and demographic groups.
**3.2 All other groups lumped in others are a small percentage, raising concerns about the results.**

We acknowledge that the "others" category, which includes a diverse range of occupations such as company employees, self-employed individuals, freelancers, and teachers, represents a small percentage of our sample. This limited representation may affect the robustness of our findings and the ability to draw meaningful conclusions about these groups.

To address this concern, we have conducted additional analyses to examine the risk perception and social support scores within the "others" category (Table 4). The results show that this group exhibited a range of risk perception and social support levels, but due to the small sample size, the statistical power of these analyses is limited. We have included these findings in the revised manuscript and discussed the implications in the "Discussion" section.

We have also emphasized in the "Limitations" section that the small sample size of the "others" category limits the generalizability of our findings to these occupational groups (Lines 218-223). We suggest that future studies should use stratified sampling methods to ensure adequate representation of diverse occupational groups, which would provide more robust insights into the risk perception and social support dynamics across different professions.

We have revised the relevant sections of our manuscript to reflect these changes and have included detailed explanations and additional analyses to address these limitations. Please find the revised manuscript attached for your review.

**4. Minor comments:**

**4.1 Further details about the validation of the risk perception scale need to be added in addition to the reference.**

We sincerely thank the reviewers for their constructive feedback. In the article (Lines 111-127), we explain in detail the development and validation process of the Risk Perception Scale (RPS) : the Risk Perception Scale (RPS) was developed based on a comprehensive literature review and theoretical framework to ensure cultural specificity and multidimensional risk structure. The scale was validated through a multi-stage process, including content validity assessment by 24 public health and psychology experts, exploratory factor analysis (EFA) with a sample of 200 individuals, and reliability testing. The validation process included individuals aged 12-70 years, ensuring that the scale is appropriate for both children and adults. We have detailed the validation process in the "Instruments" section and provided additional references to support the scale's validity across different age groups.

We have revised the relevant sections of our manuscript to reflect these changes and have included detailed explanations and references. Please find the revised manuscript attached for your review.

**Reviewer #4**

Thank you for your insightful and constructive feedback on our manuscript. We have carefully reviewed your comments and have made the necessary revisions to address each point. Below is our detailed response to the specific issues you raised:

**1. The introduction provides some epidemiological context, but it lacks a clear rationale for focusing on Chengdu. Why was this city selected? Was Chengdu more severely affected by influenza resurgence than others? Is it representative of a broader population, or does it have unique sociocultural traits relevant to risk perception and support systems?**

We have added a detailed rationale for selecting Chengdu in the "Introduction" section. Chengdu was chosen for several reasons (Lines 67-75):

1.1 Epidemiological Significance: Chengdu experienced a significant resurgence in influenza cases post-COVID-19, with a 37% higher influenza hospitalization rate compared to the national average in 2023. This makes it an ideal location to study the dynamics of influenza risk perception and social support in the context of a major public health challenge.

1.2 Diverse Population: Chengdu is a large urban center with a diverse population, including a wide range of occupations and socioeconomic backgrounds. This diversity provides a rich context for studying risk perception and social support across different demographic groups.

1.3 Unique Sociocultural Context: Chengdu has a strong collectiveist culture and extensive family support networks, which are relevant to understanding how social support systems influence risk perception. These cultural traits are particularly pertinent to the study of health behaviors and risk perception in the context of infectious diseases.

We have included these points in the revised manuscript to provide a clearer rationale for our choice of study location.

**2. The use of convenience sampling introduces a significant selection bias, especially given the skewed age distribution reported (65.7% were under 25). This dramatically limits generalizability and raises concerns about the study’s ability to reflect the broader Chengdu population's perceptions. The sample also disproportionately includes healthcare workers (44.1%) and students (37.9%), suggesting recruitment was heavily tied to academic or medical institutions, which is not representative of the general community.**

We acknowledge the limitations of convenience sampling and the resulting demographic imbalances in our sample. To address these concerns, we have:

2.1 Conducted Subgroup Analyses: We performed additional subgroup analyses to compare risk perception and social support scores across different age groups and occupations (Table 4). These analyses show that while younger adults and healthcare workers exhibited distinct patterns, the overall trends and correlations remain consistent across the sample.

2.2 Discussed Implications: We have added a detailed discussion of the implications of our sampling method in the "Limitations" section, emphasizing the need for more representative sampling in future studies (Lines 209-228).

2.3 Suggested Future Directions: We propose using stratified sampling methods in future research to ensure a more balanced representation of different demographic groups and occupations (Lines 232-237).

We have revised the "Limitations" and "Future Directions" sections to reflect these changes and provide a more comprehensive discussion of the implications of our sampling method.
**3. Using a researcher-developed instrument for measuring risk perception—particularly when validated tools already exist—requires a comprehensive justification. While the authors cite high Cronbach’s alpha and content validity, these indicators alone are insufficient.**

We have provided a more comprehensive justification for using the researcher-developed Risk Perception Scale (RPS) in the "Instruments" section (Lines 111-127). The RPS was developed to address specific limitations of existing tools, particularly in the context of Chinese culture and the multidimensional nature of risk perception. Key reasons for developing this instrument include:

3.1 Cultural Specificity: Existing risk perception scales often lack cultural specificity, which is crucial for accurately measuring risk perception in the Chinese context. The RPS was developed with input from local experts and validated in a Chinese population to ensure cultural relevance.

3.2 Multidimensional Structure: The RPS includes four dimensions (risk familiarity, controllability, severity, and susceptibility) to provide a comprehensive assessment of risk perception. This multidimensional approach is essential for understanding the complex interplay between different aspects of risk perception.

3.3 Validation Process: The scale development involved three key steps:1) Content validity was confirmed by expert review (24 specialists), showing strong I-CVI (0.72-1.00) and S-CVI/Ave (0.99) scores. 2) Reliability testing demonstrated high internal consistency (α=0.822) and test-retest reliability (ICC=0.85) across 100 participants. 3) Structural validation through factor analysis led to removing 2 low-loading items (<0.5), resulting in an optimized 18-item scale.

We have included detailed information on the development and validation process of the RPS in the revised manuscript to provide a more comprehensive justification for its use.

We have revised the relevant sections of our manuscript to reflect these changes and have included detailed explanations and additional analyses to address your concerns. Please find the revised manuscript attached for your review.

Once again, we thank each reviewer for their careful consideration and valuable feedback. Your insights have greatly contributed to enhancing the completeness and quality of our manuscript. We look forward to your positive response.

Best regards,

Xianqiong Feng
