# Supplementary material for: Social support and risk perception of influenza among Chengdu residents: A cross-sectional study during post-pandemic recovery
Source: PLoS One. 2025 Aug 29;20(8):e0331052. doi: 10.1371/journal.pone.0331052 (PMC12396661; doi:10.1371/journal.pone.0331052)
Supplement: Attachment — Submitted filename: Response to Reviewers.docx [file pone.0331052.s003.docx]

**Response to Reviewer**

Dear Dr. Xing-Xiong An and Reviewers,

Thank you for taking the time to review our manuscript titled "Social support and risk perception of influenza among Chengdu residents: A cross-sectional study during post-pandemic recovery" and for providing such insightful and constructive feedback. We are deeply grateful for your valuable suggestions, which have significantly guided the enhancement of our work. With great enthusiasm, we have meticulously addressed each of your comments and implemented thorough revisions to improve the clarity, rigor, and impact of our manuscript. We sincerely hope that the revisions meet your expectations and strengthen the quality of our research.

| **Comment** | **Author response to comment** | **Line in the article** |
| --- | --- | --- |
| Journal Requirements:  Please review your reference list to ensure that it is complete and correct. If you have cited papers that have been retracted, please include the rationale for doing so in the manuscript text, or remove these references and replace them with relevant current references. Any changes to the reference list should be mentioned in the rebuttal letter that accompanies your revised manuscript. If you need to cite a retracted article, indicate the article’s retracted status in the References list and also include a citation and full reference for the retraction notice. | Thank you for your guidance. I have thoroughly reviewed the reference list and confirmed that all cited papers are valid and none have been retracted. The reference list is complete and accurate. | 291-409 |
| Reviewer #2: Suggestions for Minor Revision: Clarify Sampling Bias: The overrepresentation of healthcare workers and students may limit generalizability. Please elaborate more clearly in the discussion on how this may affect risk perception findings. | Thank you for your insightful comment regarding the sampling bias. We acknowledge that the overrepresentation of healthcare workers and students in our sample may limit the generalizability of our findings. In the discussion section, we have elaborated on how this bias could potentially affect the risk perception results. Specifically, healthcare workers, due to their professional training and frequent exposure to pathogens, might have a lower risk perception, which could lead to an underestimation of the general population's risk perception. Conversely, the higher risk perception among students, who are younger and possibly more aware due to recent pandemic experiences, might contribute to an overestimation. We have also highlighted the need for future studies to employ more representative sampling methods, such as stratified random sampling and multi-channel recruitment, to capture a broader demographic spectrum and enhance the generalizability of the findings. | 222-240 |
| Expand on Cultural Factors: The negative correlation between social support and risk perception is intriguing. A brief expansion on cultural or psychological interpretations would enrich the discussion. | We appreciate your suggestion to expand on cultural factors. The negative correlation between social support and risk perception is indeed intriguing. In our study, we interpret this correlation within the context of China's collectivist culture, where strong social support networks can provide a sense of collective security and reduce individuals' perceived vulnerability to influenza. This cultural context may explain why higher social support is associated with lower risk perception. We have briefly discussed this in the discussion section and suggest that future research could further explore how cultural norms and values influence the relationship between social support and health-related risk perception. | 193-208 |
| Minor Language Edits: Minor corrections are recommended for grammatical polishing. For example: "women 602" → should be "female: 602 (85.0%)" | Thank you for your feedback. I have made the following modifications to the manuscript in response to your suggestions:  1. Corrected the term "women 602" to "female: 602 (85.03%)" for consistency and clarity.  2. Standardized the terminology throughout the manuscript (e.g., "healthcare workers" instead of "healthcare professionals", "utilization" instead of "utilisation").  3. Polished the language for smoother flow and standard academic grammar, including consistent formatting of p-values and statistical symbols.  I believe these changes have improved the overall quality of the manuscript. Thank you again for your valuable input. | Table 1  Table 3  Table 4  Line 110,112  Line 166  Line 176-178 |
| Replace "thesis writing" in author contributions with "manuscript preparation" for more appropriate academic terminology. | Thank you for your attention to detail. We have replaced "thesis writing" with "manuscript preparation" in the author contributions section to reflect more appropriate academic terminology. | 283-284 |
| Reviewer #4: The phrasing "we assessed dietary patterns" is broad and lacks scientific precision. It does not specify which dietary patterns were under investigation (e.g., Mediterranean, fast food-heavy, plant-based), nor does it clarify the health risks measured—whether these were physiological (e.g., BMI, blood pressure) or behavioral (e.g., physical inactivity). | Thank you for the opportunity to revise our manuscript and for the thoughtful feedback provided by the reviewers. We have carefully considered all comments. Regarding the three specific points raised about dietary patterns, assessment tools, and non-communicable disease conclusions, we respectfully wish to clarify that these elements do not align with our study's scope or content. Our research exclusively examines: ①Influenza risk perception dimensions (familiarity, controllability, severity, susceptibility); ②Social support dynamics (SSRS scale); ③Their interplay in post-pandemic Chengdu.  To address the comments directly:  1. Dietary Patterns & Health Risks:  We thank the reviewer for this methodological consideration. Our study does not investigate dietary patterns or nutrition-related health risks. The focus remains strictly on influenza-specific risk perceptions (e.g., perceived severity of influenza outcomes). The term "dietary patterns" does not appear in our manuscript.  2. Dietary Assessment Tools:  We appreciate the reviewer's emphasis on validation. Our methods explicitly detail two instruments: ①Social Support Rating Scale (SSRS) (validated); ②Researcher-developed Risk Perception Scale (RPS) (psychometrically validated for influenza). No dietary assessment tools were used, as they fall outside our research objectives.  3. Non-Communicable Disease Conclusions:  We acknowledge the importance of cautious inference. Our conclusions solely pertain to influenza prevention behaviors and risk perception gaps. We make no claims about non-communicable diseases (e.g., diet-related conditions), as these were neither studied nor analyzed.  We recognize these comments reflect rigorous methodological scrutiny, and we sincerely regret any confusion that may have arisen. Should the reviewers have concerns related to our actual focus—particularly regarding our influenza risk perception scale, social support measures, or demographic analyses—we would be grateful for the opportunity to address them.  Thank you again for your stewardship of this process. We remain fully committed to enhancing this work through constructive feedback on its true substantive focus. | NA |
| Manuscript does not describe whether any validated dietary assessment tools were used. For example, was a food frequency questionnaire (FFQ) utilized? If yes, was it culturally adapted and validated in the population of interest? Without this information, it is diffcult to judge the accuracy and reliability of the dietary data. |  |  |
| Data may indicate certain unhealthy dietary patterns, the leap to concluding "significant risk of non-communicable diseases" is not supported by any inferential statistical analysis or longitudinal evidence. Cross-sectional data can suggest correlations but not causations or future health outcomes. |  |  |

Once again, we extend our heartfelt gratitude for your meticulous review and the invaluable, constructive feedback you have provided. Your insightful comments have illuminated the path for us to enhance our research comprehensively. We are confident that the rigorous revisions we have undertaken have significantly elevated the academic quality of our study, deepened its research dimensions, and broadened its scope. This has enabled our work to offer more valuable insights and references to the relevant fields. We look forward to your further evaluation of the revised manuscript and hope that our research findings can be published in your esteemed journal to contribute to the academic community. Thank you once again for your kind support and guidance!

Best regards,

Cheng Yang

2025.6.24
