# Supplementary material for: Social support and risk perception of influenza among Chengdu residents: A cross-sectional study during post-pandemic recovery
Source: PLoS One. 2025 Aug 29;20(8):e0331052. doi: 10.1371/journal.pone.0331052 (PMC12396661; doi:10.1371/journal.pone.0331052)
Supplement: Attachment — Submitted filename: Response_to_Reviewers_auresp_3.docx [file pone.0331052.s004.docx]

**Response to Reviewer**

Dear Dr. Xing-Xiong An and Reviewer 4,

Thank you for taking the time to review our manuscript titled "Social support and risk perception of influenza among Chengdu residents: A cross-sectional study during post-pandemic recovery" and for providing such insightful and constructive feedback. We are deeply grateful for your valuable suggestions. We have carefully addressed your opinions and made comprehensive revisions. We sincerely hope that these revisions will meet your expectations and enhance the quality of our research.

| **Comment** | **Author response to comment** | **Line in the article** |
| --- | --- | --- |
| Reviewer #4: Although it is good manuscript and author has addressed past comments. In some of the area still minor work needed. Such as in abstract numbers are provided (e.g., median scores, percentages, p-values), there’s no interpretive glue connecting them. For instance, the sentence: “Median social support and risk perception scores were 37 (63.79%) and 36 (40.0%) respectively” lacks context — is 40% alarmingly low? Is 63.79% moderate or high?  But overall this is acceptable. | Thank you for your helpful feedback. In response to your concern that the abstract lacked “interpretive glue,” we have added explicit qualifiers to every numeric finding. Specifically:  1. The median social-support score is now labeled “moderate” (37 points; 63.79 % of the maximum).  2. The median risk-perception score is described as “alarmingly low” (36 points; 40.0 % of the maximum) and is further contextualized with severity and familiarity sub-scores rated as “very low” (32 %) and “low” (36.7 %), respectively.  3. All percentages, p-values, and correlation coefficients now include brief interpretive phrases so readers can immediately grasp their practical significance.  We have incorporated these changes into the revised abstract and uploaded the updated manuscript. Thank you again for guiding us toward a clearer presentation. | 28-39 |

Thank you again for your constructive comments and for shepherding the manuscript toward acceptance.

Best regards,

Cheng Yang

2025.8.7
